# Circular Single-Stranded Synthetic DNA Delivery Vectors for MicroRNA

**DOI:** 10.1371/journal.pone.0016925

**Published:** 2011-02-16

**Authors:** Christine I. Seidl, Kevin Ryan

**Affiliations:** 1 Department of Chemistry, City College of New York, New York, New York, United States of America; 2 The Graduate Center, City University of New York, New York, New York, United States of America; Institut National de la Santé et de la Recherche Médicale, France

## Abstract

Single-stranded (ss) circular oligodeoxynucleotides were previously found to undergo rolling circle transcription (RCT) by phage and bacterial RNA polymerases (RNAPs) into tandemly repetitive RNA multimers. Here, we redesign them to encode minimal primary miRNA mimics, with the long term aim of intracellular transcription followed by RNA processing and maturation via endogenous pathways. We describe an improved method for circularizing ss synthetic DNA for RCT by using a recently described thermostable RNA ligase, which does not require a splint oligonucleotide to juxtapose the ligating ends. In vitro transcription of four templates demonstrates that the secondary structure inherent in miRNA-encoding vectors does not impair their RCT by RNAPs previously shown to carry out RCT. A typical primary-miRNA rolling circle transcript was accurately processed by a human Drosha immunoprecipitate, indicating that if human RNAPs prove to be capable of RCT, the resulting transcripts should enter the endogenous miRNA processing pathway in human cells. Circular oligonucleotides are therefore candidate vectors for small RNA delivery in human cells, which express RNAPs related to those tested here.

## Introduction

Small RNAs such as miRNA and siRNA are now well established as sequence-specific regulators of gene expression at the mRNA level [1]. For experimental and potential therapeutic uses, small RNAs must either be synthesized and introduced into cells directly [2], [3], or encoded by double stranded (ds) DNA vectors whose transcripts are made and processed within a cell [4], [5], [6]. While useful and effective, neither approach is without drawbacks. RNA is more expensive, more difficult to synthesize and less stable in storage than DNA, and ribonucleases from serum, cellular or other sources can degrade small RNAs before they reach their target. Plasmid and viral vectors encoding short hairpin transcripts (shRNA) carry the same information in relatively stable form, but typically waste more than 99% of their nucleotide (nt) mass. They must also be biosynthesized in cells or by microorganisms, risking contamination in therapeutic applications, and can undergo genomic integration, potentially causing cancer [7]. These shortcomings in small RNA delivery methods have prompted us to investigate single stranded circular oligodeoxynucleotides (COLIGOs) as minimized relay molecules for shuttling small RNA sequence information into cells.

**Figure 1:**
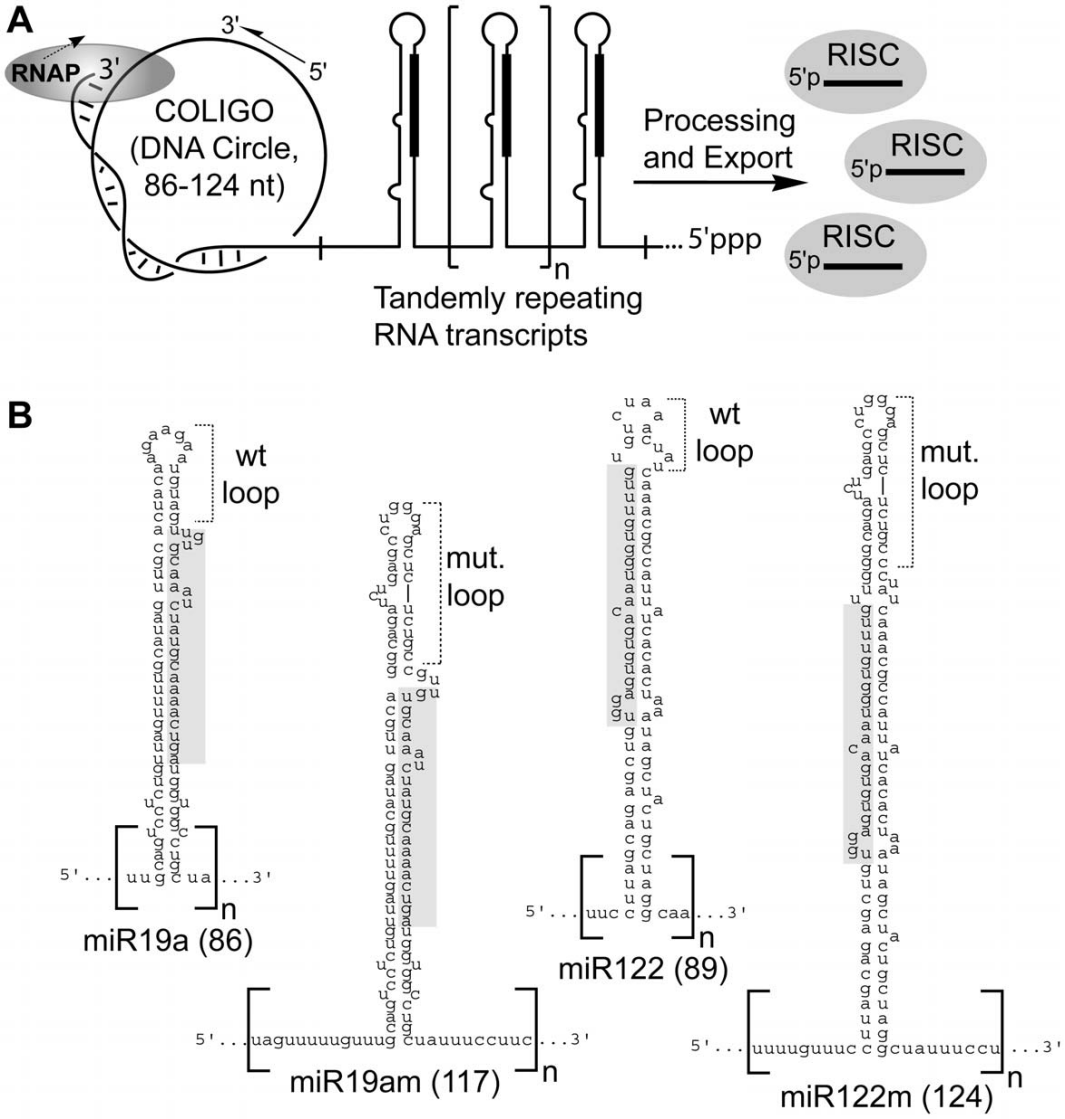
Rolling Circle Transcription (RCT) approach to expressing miRNA, siRNA and other small RNA. **A**. Circular oligodeoxynucleotides (COLIGOs) are made to encode minimal pri-miRNAs. RCT would produce tandemly arrayed primary miRNA stem-loops resembling naturally occurring miRNA clusters to promote entry into the endogenous miRNA maturation pathway, and Argonaute effector complexes (RISC) programming. **B**. RNA transcript sequences and the predicted secondary structures of the four COLIGOs used in this study. Mature miRNA are shaded, number in parentheses refers to number of nucleotides in COLIGO and its monomeric transcript, which is shown arbitrarily beginning outside of the stem-loop.

Single-stranded (ss) DNA circles were previously found to serve as *in vitro* templates for bacteriophage RNA polymerases (RNAPs) [8] and later *E. coli* RNAP [9], [10], [11]. Lacking a promoter, they undergo relatively infrequent transcription initiation but, once engaged, possibly by virtue of a resemblance to the transcription bubble, their circular topology permits transcription around the template many times, a process termed rolling circle transcription, or RCT (Fig. 1A). RCT can lead to the production of large multimeric transcripts before the RNAP terminates. Although their size (∼20–200 nt) limits the amount of information they can carry, COLIGOs are well suited to encoding the amount of information found in small RNAs, as previously demonstrated for ribozyme sequences [9], [11]. If the multimeric transcript can be made to undergo processing, for example self-processing in the ribozyme case, a large amplification of the DNA sequence into many small RNA copies per transcription event can result. This approach has been used recently as an *in vitro* alternative to run-off transcription in the production of siRNA using T7 RNAP [12].

We are investigating the possibility that COLIGOs might be useful as small RNA intracellular delivery vectors – chemically synthesized like siRNA but constructed in circular form from DNA, and thus more chemically stable. COLIGOs are resistant to exonuclease activity and easier to synthesize free of biologic contamination than shRNA-encoding plasmids and viruses. In addition, we envision the possibility that COLIGOs would give rise to a multimeric transcript that, by mimicking transcripts formed during the expression of endogenous miRNA clusters [13], would enter the natural miRNA maturation pathway, ultimately programming ribonucleoprotein complexes capable of miRNA and siRNA activity (Fig. 1A). To begin an assessment of this approach for producing small RNA in cells, it is necessary to test whether COLIGOs encoding primary (pri)-miRNA transcripts are accepted as transcription templates by RNAPs, and to learn whether RCT RNA multimers will be processed by Drosha/Microprocessor, the maturation pathway entry point leading to miRNA. In addition to describing an improved method for circularizing the linear RCT template oligonucleotides, we show here that miRNA-encoding COLIGOs are substrates for RCT by bacterial and bacteriophage RNAPs. Our additional finding that miRNA rolling circle transcripts are accurately processed by the human Drosha/Microprocessor complex provides impetus for future studies assessing the RCT capabilities of human RNAPs.

## Results

### Design and synthesis of circular DNA templates encoding miRNAs

We designed DNA circles to encode shortened versions of two human pri-miRNAs, miR-19a and miR-122. We based our design on the endogenous human miRNA genomic template-strand sequences, and included DNA encoding ∼3 helical turns of the pri-miRNA stem-loop sequence (Fig. 1B), the minimum stem length required for Drosha/Microprocessor processing [14], [15]. In two of the COLIGOs we minimized the length of the sequences flanking the stem-loops to 4 nt (COLIGO 19a) and 6 nt (COLIGO 122) per repeat. In two other COLIGOS, 19am and 122m, we included more of the natural flanking sequences (taken in both cases from the miR19a genomic template-strand sequence), for a total of 22 and 18 flanking nt, respectively. Additional flanking nt are believed to enhance Drosha processing [15], [16]. However, in a rolling circle transcript all but the terminal stem-loop repeats would be flanked by varying lengths of RNA, so it is not yet clear how much flanking RNA should be encoded by a given COLIGO. The complete monomer transcripts for the four templates - two for each miRNA - used in this study are shown in Fig. 1B. Although the pri-miRNA stem-loop structure forms the basis of our COLIGO design, we note that promoterless transcription may initiate at multiple sites in the DNA circle, and that alternate folding patterns are possible for multimeric transcripts [12].

Some miRNAs have conserved loops that aid in their processing [17], but specific loop sequences are likely not critical to the biogenesis of most miRNAs [15], [18]. Loop sequences at the DNA level in the COLIGO, however, may influence transcription efficiency, as shown for *E. coli* RNAP RCT [19]. We tested this variable, in combination with the longer flanking sequences, by swapping the native miRNA loops in pre-miR-19a and miR-122 for an unrelated RNA hairpin loop (from HIV TAR RNA), resulting in pre-miR19am and pre-miR122m (monomer transcripts are shown in Fig. 1B).

To choose the site where the linear oligonucleotide is closed to form the COLIGO we predicted the folding of the COLIGO using mFold [20] and selected the region having the least secondary structure, which in the linear form might prevent juxtaposition of the ends. This sequence typically coincided with the flanking nucleotides at the base of the stem (of the transcripts). The DNA circles encoding the transcripts shown in Fig. 1B were therefore ligated between the 5′ and 3′ deoxynucleotides corresponding to the 3′ and 5′ terminal ribonucleotides, respectively, depicted in the monomer transcripts (Fig. 1B).

Rolling circle transcription templates have been circularized by three methods: chemical ligation within a DNA triple-helix [8]; T4 DNA ligase closure mediated by a splint oligonucleotide [21]; and T4 DNA ligase closure within a nicked DNA dumbbell structure, as for the shRNA-encoding templates [12]. Among these methods, splinted DNA ligase closure should be the most general approach for natural miRNA cDNAs, since their stem sequences include multiple mismatches, which might interfere with DNA ligase closure. However, the requirement for a splint to bring the 5′ and 3′ ends together increases the cost of DNA synthesis and requires an additional step to remove the splints after circularization. In preliminary experiments we also found that splint-mediated ligation resulted in unsatisfactory circularization yields and varying amounts of linear multimers.

**Figure 2:**
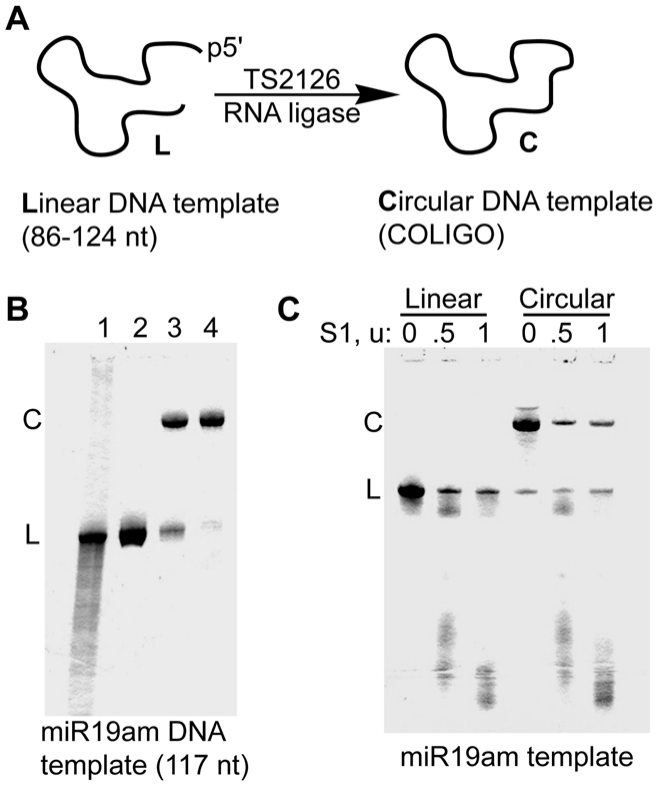
Circularization of DNA templates (COLIGOs) for Rolling Circle Transcription. **A**. Synthetic 5′ phosphorylated linear DNA sequences were circularized using the thermostable TS2126 RNA ligase. **B**. Denaturing polyacrylamide gel electrophoresis (DPAGE) at four stages during miR-19am DNA circle synthesis. Lane 1, crude DNA IDT Ultramer synthesis of COLIGO 19am. Lane 2, after preparative DPAGE. Lane 3, crude circularization product. Lane 4, DNA circle template following Exonuclease I clean-up. Visualization using Stains-All. **C**. Verification of circular topology. Nicking of circular templates by S1 nuclease leads first to linear forms, which are then further digested to successively smaller linear forms.

We obtained better results using a newly discovered thermostable RNA ligase (Rnl) from bacteriophage TS2126 (Fig. 2A). TS2126 Rnl has been reported to efficiently circularize ss DNA without a splint [22]. In a typical COLIGO preparation using this enzyme, the entire 5′ phosphorylated linear template was made in one synthesis, gel purified by DPAGE and circularized with TS2126 Rnl. Most residual linear form was then removed by Exonuclease I treatment. For example, in the case of the 117 nt template encoding the miR19am transcript (Fig. 1B), 5.0 nmol from the desalted DNA synthesis was gel purified to yield 1.2 nmol of the full-length form (23% full-length recovered). Following cyclization and Exonuclease I treatment, 0.87 nmol of the COLIGO was recovered (73% cyclization based on purified linear precursor). The gel profile of the DNA at the various stages is shown in Fig. 2B. A final prep-scale gel purification generally does not improve the circular-to-linear ratio, and leads to a large loss of COLIGO. The circular topology of all COLIGOs used in this study was verified by S1 nuclease nicking (e.g. Fig. 2C), which for COLIGOs leads initially to the linear form with no intermediate gel bands [21].

### In vitro transcription by bacteriophage and bacterial RNA polymerases

**Figure 3:**
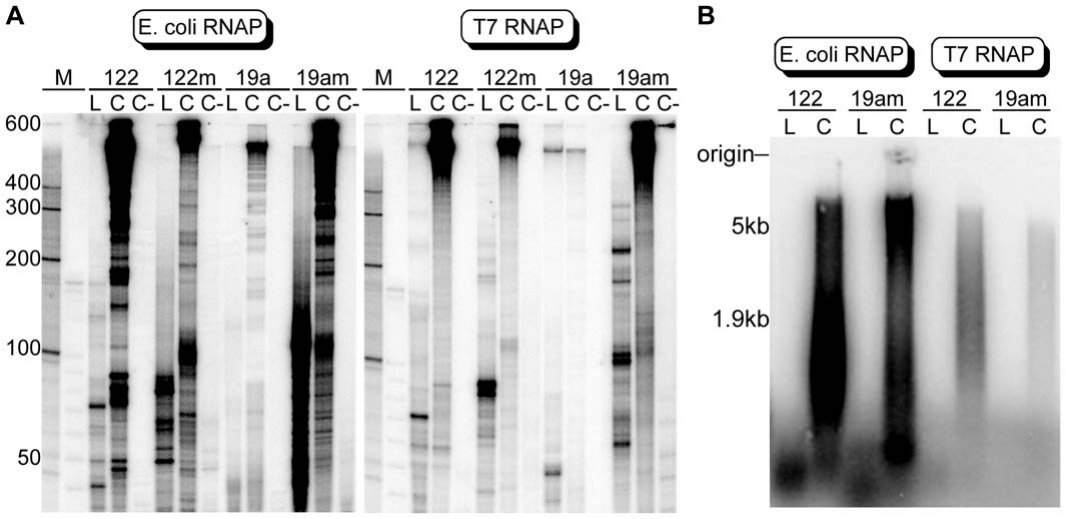
*In vitro* transcription of miRNA-encoding COLIGOs by *E. coli* and bacteriophage T7 RNA polymerases. **A**. Linear (L) and circular (C) templates 122, 122m, 19a, 19am, encoding either pre-miR-122 or pre-miR-19a, were transcribed in the presence of all NTPs or all except ATP (C-). 9% DPAGE. The Phosphorimager sensitivity setting was increased 5-fold for the weaker T7 RCT reactions. **B**. Denaturing 1% agarose gel showing the high molecular weight size range of the transcripts. No adjustment in the Phosphorimager sensitivity setting was made.

While COLIGOs encoding shRNAs [12] and ribozymes [9], [10], [11] have been shown to undergo RCT *in vitro* by bacterial and bacteriophage RNAPs, COLIGOs encoding native pri-miRNAs have not been investigated. To determine whether native miRNA cDNA secondary structure might pose a general impediment to RCT, we exposed the four COLIGOs encoding miR-19a and miR-122 to T7 and *E. coli* RNAPs *in vitro* (Fig. 3A). Transcripts were visualized by [α-^32^P]-UTP incorporation. All four COLIGOs generated the large transcripts characteristic of RCT. For each template sequence, the circular topology was required to produce transcripts significantly longer than the COLIGOs' circumference, and withholding one nucleotide triphosphate (ATP, Lane C-) led as expected to the loss of all but very short aborted transcripts. For all templates, more RNA was produced using *E. coli* RNAP compared to T7 RNAP under similar conditions (see Materials and Methods). Denaturing formaldehyde agarose gel electrophoresis showed that the maximum length of the transcripts typically reached 5 kilobases (5 kb, Fig. 3B). Thus, all four DNA circles served as RCT templates despite the extensive secondary structure predicted for COLIGOs encoding pri-miRNA stem loop structures.

The relative amount of RNA produced in these experiments varied with the DNA sequence outside of the miRNA stem sequence. The template encoding the pre-miR-19a with the natural loop and short flanking sequence was noticeably poor (Fig. 3A), but it became a more efficient template when its wild-type (wt) loop was replaced with the TAR RNA loop and the flanking sequences were lengthened (compare 19a and 19am). In contrast, the same loop and flanking sequences, when placed on the miR122 stem, decreased transcription efficiency slightly (compare 122 and 122m). These comparisons demonstrate that the non-essential sequence regions of a pri-miRNA-encoding RCT template can influence the extent to which a COLIGO is transcribed.

### Drosha/Microprocessor processing of rolling circle transcripts

**Figure 4:**
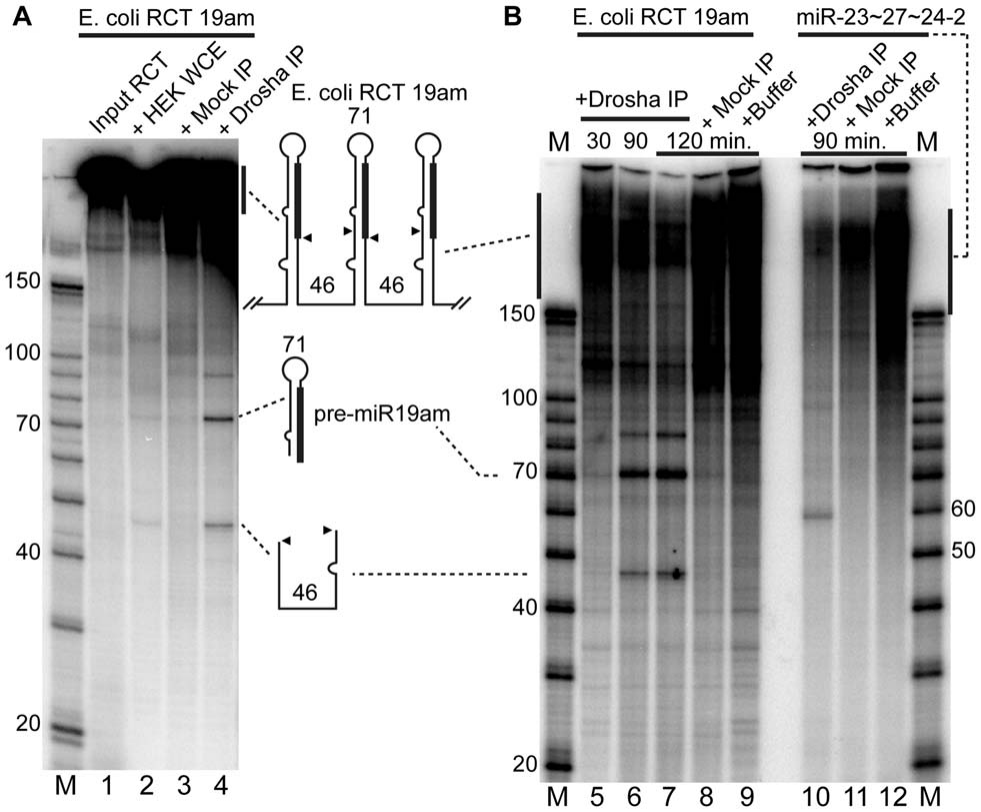
*In vitro* Drosha processing of miRNA Rolling Circle Transcripts (RCT). **A**. Deproteinized miR-19am RCTs made *in vitro* using *E. coli* RNA polymerase were incubated with HEK293T whole cell extract (WCE, Lane 2); with the Flag-Drosha complex immunoprecipitated from Flag-Drosha-expressing HEK293T WCE (Lane 4); or with Flag immunoprecipitate from WCE made from untransfected HEK293T cells (Mock, Lane 3). 90 min. processing reactions. **B**. Drosha processing reactions at various processing times using DPAGE gel purified miR-19am RCT transcripts (lanes 5–9) or the miR-23a∼27a∼24-2 cluster transcript as a positive control (lanes 10–12) at the standard 90 min. processing reaction time.

Primary miRNA transcripts are first processed in their natural stem-loop context by Microprocessor, a complex that in mammalian cells includes Drosha and DGCR8 [23], [24]. We tested whether pre-miRNA hairpins could be processed from a multimeric rolling circle transcript. Treatment of RCT transcripts (made *in vitro* with uniform ^32^P-labeling using *E. coli* RNAP) with HEK293T whole cell extract (WCE) led to the release of low levels of the pre-miRNA hairpin (71 nt predicted) and the intervening flanking region (46 nt predicted) (Fig. 4A, lane 2). In order to test whether Drosha was responsible for this processing, we immunoprecipitated (IP'd) FLAG-tagged Drosha from transfected HEK293T cells [16]. The activity of IP'd Drosha (and co-IP'd proteins) was verified on an *in vitro* transcribed human miRNA cluster whose processing is predicted to produce three pre-miRNAs of 57-nt (pre-miR-23a), 59-nt (pre-miR-24-2) and 62-nt (pre-miR-27a) [13] (Fig. 4B, lane 10, ∼60 nt RNA was produced). When the miR-19a RCT transcript was treated with IP'd Drosha, the same processed RNAs produced by WCE were released from the transcript, but in higher amounts (Fig. 4A, lane 4). The processed transcripts accumulated over the standard *in vitro* Drosha processing time period of 90 minutes (Fig. 4B, lanes 5, 6, 7), making it unlikely that they resulted from random degradation. These results demonstrated that pre-miRNA hairpins can be accurately processed by human Drosha from RCT transcripts. If RCT were to take place in the nucleus of human cells, then some of the RNA should be capable of entering the natural processing pathway.

## Discussion

Naturally occurring small RNAs have emerged in recent years as important regulators of cellular biochemistry. Small RNAs of designed sequence are regularly used to investigate gene function, and hold great promise for therapeutic intervention. However, the problem of delivering the RNA to the inside of a human cell remains an obstacle to their therapeutic exploitation. We are attempting to recast this problem into a potentially more tractable one, wherein a small chemically synthesized DNA sequence, in circular form, is used to carry the information for making the small RNA into a cell. Our approach could result in a distinct alternative to siRNA and shRNA vectors.

The work presented here demonstrates a new enzymatic method for circularizing synthetic DNA oligonucleotides for use in RCT. In addition, we find that the secondary structure necessarily present in a COLIGO encoding pri-miRNAs does not impair RCT by bacterial and bacteriophage RNAP. Both of these RNAPs are related to eukaryotic RNAPs. The T3 and T7 bacteriophage RNAPs have significant sequence homology to the mitochondrial (mt) RNAP [25], a splice variant of which was recently found to be expressed in the nucleus of human cells [26]. The three major eukaryotic nuclear RNAPs – I, II and III – contain subunits with sequence and structural homology to the bacterial enzyme's α and β subunits [27]. Coupled with our finding that the human Drosha/Microprocessor complex processes rolling circle transcripts, this homology justifies future, though more technically difficult, testing of human RNAPs for RCT on COLIGOs designed according to the strategy described here.

## Materials and Methods

### Materials, reagents and general procedures

Synthetic DNA was made by Integrated DNA Technologies (Coralville, IA) and chemically 5′ phosphorylated. Preparative and analytical denaturing polyacrylamide gel electrophoresis (DPAGE) and denaturing agarose electrophoresis were done according to standard procedures [28]. DNA was located on preparative gels using UV shadowing over fluorescent silica gel plates (EMD Chemicals 5715-7). DNA was recovered by electroelution in 1xTBE within a sealed dialysis membrane tube with a molecular weight cut off of 1000 (Spectrum Laboratories). Eluted DNA was phenol-chloroform extracted and ethanol precipitated with sodium acetate. In some cases, the DNA gel slice was eluted in 0.3 M sodium-acetate at 37°C overnight and ethanol precipitated. The 1% agarose gel was prepared using Ambion's 10X denaturing gel buffer, blotted overnight onto positively charged nitrocellulose (Ambion), baked for 20 min at 55°C, UV crosslinked and exposed to a PhosphorImager® screen (Molecular Dynamics). *E. coli* RNAP was purchased from USB. T7 RNAP and DNA ligase were purchased from New England Biolabs. RNA markers were purchased from Sigma (R4142) or Ambion (Decade marker, AM7778) and prepared according to the manufacturer's instructions. For the agarose gel (Fig. 3B), sizes were determined by running 8 µg of total cellular RNA extracted from HEK293T cells on the same gel and staining for ribosomal RNA (18S = 1.9 kb and 28S = 5 kb) with methylene blue after blotting.

### Synthesis of linear DNA templates

Linear DNA templates encoding shortened forms of pri-miR-19a, -miR-122 and -miR19am (e.g. Fig. 2B) were synthesized by IDT as single 5′ phosphorylated Ultramer sequences. The linear templates for miR122m and miR19am were also made from two half-length oligonucleotides (e.g. 19am-1 and 19am-2) according to the standard splint mediated T4 DNA ligase procedure [21]. The following sequences were used, including splint oligonucleotides where applicable:

**19am** (117 nt; when the linear form was synthesized as one sequence, it began as in 19am-1 and continued through 19am-2): 19am-1 (63 nt): 5p-GAAGGAAATAGCAGGCCACCATCAGTTTTGCATAGATTTGCACAACGGCAGAGAGCTCCCAGG; 19am-2 (54 nt): 5′p-CTCAGATCTGCCTGCAACTATGCAAAACTAACAGAGGACTGCAAACAAAAACTA, splint 19am (30 nt): 5′-CAGGCAGATCTGAGCCTGGGAGCTCTCTGC; **122m** (124 nt): 122m-1 (65 nt): 5′p- AGGAAATAGCCTAGCAGTAGCTATTTAGTGTGATAATGGCGTTTGATAGGGCAGAGAGCTCCCAG, 122m-2 (59 nt): 5′p-GCTCAGATCTGCCCACAAACACCATTGTCACACTCCACAGCTCTGCTAAGGAAACAAAA, splint 122m (31 nt): 5′-TGGGCAGATCTGAGCC-TGGGAGCTCTCTGCC; **19a** (86 nt): 5′p-TAGCAGGCCACCATCAGTTTTGCATAGATTTGCACAACTACATTCTTCTTGTAGTGCAACTATGCAAAACTAACAGAGGACTGCAA; **122** (89 nt) 5′p-TTGCCTAGCAGTAGCTATTTAGTGTGATAATGGCGTTTGATAGTTTAGACACAAACACCATTGTCACACTCCACAGCTCTGCTAAGGAA.

### TS2126 RNA Ligase I

A synthetic gene (Top Gene Technologies Inc., Canada) encoding the first 393 amino acids of TS2126 RNA ligase (Rnl) I [22] followed by a C-terminal hexahistidine tag was inserted into the EcoRI/SmaI sites of pBluescriptSK(+) plasmid (Stratagene). The synthetic DNA contained codons optimized for expression in *E. coli*. The entire coding sequence of this plasmid, pTS2126H, was verified by DNA sequencing. *E. coli* Bl21-CodonPlus(DE3)-RIL (Stratagene) was transformed with pTS2126H and grown in 2 liters of culture. At OD_595_ ∼0.5 the culture was induced to a final concentration of 1 mM IPTG and grown for another 3 hrs at 37°C. The cells were pelleted at 3000 g for 15 min at 4°C, resuspended in 30 ml native lysis buffer (50 mM NaH_2_PO_4_, 300 mM NaCl, 10 mM imidazole, pH 8 with NaOH), and disrupted using a French press. After centrifugation to remove debris, the lysate was incubated with 1 ml Ni-NTA agarose (Qiagen) with agitation for 2 hrs at 4°C. The beads were washed three times with 5 ml native lysis buffer each time, once with elution buffer without imidazole (20 mM Tris-HCl pH 8, 100 mM KCl, 2 mM DTT) and finally eluted with 500 µl portions of elution buffer containing 250 mM imidazole. The pooled fractions containing the protein were dialyzed (3 times, 2 hrs each) against 10 mM Tris-HCl (pH 8), 50 mM KCl, 0.1 mM EDTA and 1 mM DTT, and stored at 4° C (short term) or in 50% glycerol and –80°C (long term).

### Circularization of oligonucleotides

Reactions contained a ratio of 0.015 nmol linear 5′ phosphorylated DNA (0.75 µM final conc.) to 1.5 µg TS2126 Rnl and the following components (final concentrations): 50 µM ATP, 2.5 mM MnCl_2_, 50 mM MOPS (pH 7.5), 10 mM KCl, 5 mM MgCl_2_ and 1 mM DTT. These conditions were used for reactions ranging from 015 to 1.5 nmol. Circularizations were incubated for 2 hrs at 60°C, followed by phenol/chloroform extraction and ethanol precipitation. In preliminary syntheses, the circularized products and unreacted linear templates were separated on a 1.5 mm DPAGE and visualized by UV shadowing, excised from the gel and electroeluted. In cases where the COLIGO was still contaminated by >5% of the linear oligonucleotide after elution (as determined by gel staining), an Exonuclease I (NEB) digest was done. For most COLIGOs used in this study, we used the more recently adopted procedure shown in Fig. 2B. The crude desalted oligonucleotide was gel-purified, circularized as described above, phenol/chloroform extracted and precipitated. The COLIGO was then treated with 10 u of Exonuclease I per 0.1 nmol DNA in a total volume of 50 µl according to the manufacturer's instructions, phenol/cholorform extracted, precipitated and used without further gel-purification after analytical scale DPAGE showed no multimeric circles and less than 5% linear form by 0.05% Stains-All (Acros) staining.

### S1-nuclease assay

To verify the circular topology of the COLIGOs (as in Fig. 2C), the following procedure was used: a 10 µl reaction containing 1 µg of linear or circular oligonucleotide and 0, 0.5 or 1 u of S1 nuclease (USB) in 50 mM sodium acetate, pH 4.6, 1 mM zinc acetate, 250 mM NaCl and 0.5 µg BSA was incubated for 10 min at 37°C, extracted with phenol/chloroform, ethanol precipitated, separated by 10 or 12% DPAGE and visualized with Stains-All.

### *In vitro* transcription

The following conditions were used for *in vitro* transcription with bacteriophage T7 and *E. coli* RNAPs: final COLIGO concentration 1 µM, 1unit/µl RNAse inhibitor (Promega), 0.5 mM each ATP, CTP and GTP, 0.05 mM UTP in Fig. 3A and 3B; or 1 mM each NTP in Fig. 4A and 4B; ∼2 µCi [α-^32^P]-UTP; and (for T7 RNAP: 40 mM Tris-HCl pH 7.9, 6 mM MgCl_2_, 10 mM DTT, 2 mM spermidine); (for *E.coli:* 40 mM Tris-HCl, pH 8.0, 10 mM MgCl_2_, 5 mM DTT, 50 mM KCl, 50 µg/ml BSA). Each 10 µl RCT reaction (Fig. 3) contained the following amount of RNAP as defined by the vendor: 40 units T7, 1 unit *E.coli*. Reactions were incubated for 1 hr at 37°C, after which time the RNA was extracted with 150 µl TriReagent (Ambion), isopropanol precipitated according to the manufacturer's instructions with 10 µg glycogen added. For all *in vitro* RNAP reactions, radiolabeled transcripts were separated on a 9% denaturing polyacrylamide gel, dried and exposed to a PhosphorImager® screen. The T7 RNAP reactions shown in Fig. 3A, which were done on the same scale and contained the same amount of [α-^32^P]-UTP as the *E. coli* reactions in the same figure, are shown at 5 times greater sensitivity to adjust for the lower level of incorporation with the T7 enzyme. For comparison, no adjustment was made in Fig. 3B. The transcripts in Fig. 4A were, after transcription at the relatively high concentration of 1 mM cold NTP (see above), deemed sufficiently free of shorter transcripts to be used for processing without gel purification. In Fig. 4B we gel-purified the transcripts by 6% DPAGE and then eluted the RNA for 3 hrs at 37°C in 350 µl 0.5 M ammonium acetate, 1 mM EDTA, 0.2% SDS, extracted with an equal volume of phenol chloroform-isoamyl alcohol and precipitated with 0.3 volumes of 3 M sodium acetate, 2.5 volumes ethanol and 10 µg glycogen. Eluting the RNA from the gel led to some degradation, which produced a wider distribution of RCT transcript sizes in the processing input RNA in Fig. 4B. Some non-specific degradation also occurred during the processing reactions shown in Fig. 4B, since not all of the missing input is accounted for in the processed fragments.

### Drosha immunoprecipitation and *in vitro* RNA processing

The pCK-Drosha-FLAG plasmid (kindly provided by V. N. Kim) was expressed and purified as previously described [16]. Briefly, 8 µg of plasmid were transfected per 6 cm dish of HEK293T cells using 20 µl Lipofectamine 2000 (Invitrogen). After 44 hrs, the cells were lysed (900 µl Sigma FLAG® kit lysis buffer/6 cm dish) and Flag-IP was carried out using 40 µl of a 50% anti-FLAG bead slurry (Sigma FLAG® Tagged Protein Immunoprecipitation Kit) for at least 4 hrs at 4°C. The beads were washed 4 times with 1X Wash Buffer and once in processing buffer (20 mM Hepes-KOH, pH 7.9, 100 mM KCl, 0.2 mM EDTA, 0.5 mM DTT, 0.2 mM PMSF, 5% glycerol). A parallel mock-IP was carried out using Lipofectamine without the Drosha-encoding plasmid. The activity of the immunoprecipitated protein was verified using the miRNA-23a∼27a∼24-2 cluster transcribed *in vitro* from plasmid pGEM-T-easy_pri-miR-23, 27, 24-2(+) (also provided by V. N. Kim). For HEK293T WCE as the Drosha source, cells were lysed as described above; the crude lysate was agitated at 4°C for 30 min, centrifuged for 30 min at 13000 rpm and the supernatant collected and adjusted to 20% glycerol. For *in vitro* processing of rolling circle transcripts from COLIGO 19am, 1/10 of an *in vitro* transcription reaction using *E. coli* RNA polymerase (described above) or ¼ of the eluted pri-miR-23a∼27a∼24-2(+) transcript was used in a total volume of 30 µl containing 15 µl beads from Drosha Flag-IP (or mock-IP, or processing buffer including 6.4 mM final MgCl_2_ and 0.1 u/µl RNase inhibitor). The processing reaction was carried out for 90 min at 37°C (Fig. 4A), or for the times indicated in Fig. 4B. RNA was extracted with TriReagent (Ambion), isopropanol precipitated and separated on a 10% denaturing polyacrylamide gel, dried and exposed to a PhosphorImager® screen.

## References

[1] Chu CY, Rana TM (2007). Small RNAs: regulators and guardians of the genome.. J Cell Physiol.

[2] Elbashir SM, Lendeckel W, Tuschl T (2001). RNA interference is mediated by 21- and 22-nucleotide RNAs.. Genes Dev.

[3] Siolas D, Lerner C, Burchard J, Ge W, Linsley PS (2005). Synthetic shRNAs as potent RNAi triggers.. Nat Biotechnol.

[4] Manjunath N, Wu H, Subramanya S, Shankar P (2009). Lentiviral delivery of short hairpin RNAs.. Adv Drug Deliv Rev.

[5] Paddison PJ, Caudy AA, Bernstein E, Hannon GJ, Conklin DS (2002). Short hairpin RNAs (shRNAs) induce sequence-specific silencing in mammalian cells.. Genes Dev.

[6] Brummelkamp TR, Bernards R, Agami R (2002). A system for stable expression of short interfering RNAs in mammalian cells.. Science.

[7] Glover DJ, Lipps HJ, Jans DA (2005). Towards safe, non-viral therapeutic gene expression in humans.. Nat Rev Genet.

[8] Daubendiek SL, Ryan K, Kool ET (1995). Rolling-Circle RNA Synthesis: Circular Oligonucleotides as Efficient Substrates for T7 RNA Polymerase.. J Amer Chem Soc.

[9] Diegelman AM, Kool ET (1998). Generation of circular RNAs and trans-cleaving catalytic RNAs by rolling transcription of circular DNA oligonucleotides encoding hairpin ribozymes.. Nucleic Acids Res.

[10] Diegelman AM, Kool ET (1999). Mimicry of the hepatitis delta virus replication cycle mediated by synthetic circular oligodeoxynucleotides.. Chem Biol.

[11] Daubendiek SL, Kool ET (1997). Generation of catalytic RNAs by rolling transcription of synthetic DNA nanocircles.. Nat Biotechnol.

[12] Seyhan AA, Vlassov AV, Johnston BH (2006). RNA interference from multimeric shRNAs generated by rolling circle transcription.. Oligonucleotides.

[13] Lee Y, Jeon K, Lee JT, Kim S, Kim VN (2002). MicroRNA maturation: stepwise processing and subcellular localization.. Embo J.

[14] Zeng Y, Yi R, Cullen BR (2005). Recognition and cleavage of primary microRNA precursors by the nuclear processing enzyme Drosha.. Embo J.

[15] Han J, Lee Y, Yeom KH, Nam JW, Heo I (2006). Molecular basis for the recognition of primary microRNAs by the Drosha-DGCR8 complex.. Cell.

[16] Lee Y, Ahn C, Han J, Choi H, Kim J (2003). The nuclear RNase III Drosha initiates microRNA processing.. Nature.

[17] Michlewski G, Guil S, Semple CA, Caceres JF (2008). Posttranscriptional regulation of miRNAs harboring conserved terminal loops.. Mol Cell.

[18] Zeng Y, Cullen BR (2003). Sequence requirements for micro RNA processing and function in human cells.. RNA.

[19] Ohmichi T, Maki A, Kool ET (2002). Efficient bacterial transcription of DNA nanocircle vectors with optimized single-stranded promoters.. Proc Natl Acad Sci U S A.

[20] Zuker M (2003). Mfold web server for nucleic acid folding and hybridization prediction.. Nucleic Acids Res.

[21] Diegelman AM, Kool ET (2000). Chemical and Enzymatic Methods for Preparing circular Single-Stranded DNAs..

[22] Blondal T, Thorisdottir A, Unnsteinsdottir U, Hjorleifsdottir S, Aevarsson A (2005). Isolation and characterization of a thermostable RNA ligase 1 from a Thermus scotoductus bacteriophage TS2126 with good single-stranded DNA ligation properties.. Nucleic Acids Res.

[23] Han J, Lee Y, Yeom KH, Kim YK, Jin H (2004). The Drosha-DGCR8 complex in primary microRNA processing.. Genes Dev.

[24] Gregory RI, Yan KP, Amuthan G, Chendrimada T, Doratotaj B (2004). The Microprocessor complex mediates the genesis of microRNAs.. Nature.

[25] Masters BS, Stohl LL, Clayton DA (1987). Yeast mitochondrial RNA polymerase is homologous to those encoded by bacteriophages T3 and T7.. Cell.

[26] Kravchenko JE, Rogozin IB, Koonin EV, Chumakov PM (2005). Transcription of mammalian messenger RNAs by a nuclear RNA polymerase of mitochondrial origin.. Nature.

[27] Ebright RH (2000). RNA polymerase: structural similarities between bacterial RNA polymerase and eukaryotic RNA polymerase II.. J Mol Biol.

[28] Sambrook J, Russell D (2001). Molecular cloning: A laboratory manual..

